# Drivers of the progress achieved by Peru in reducing childhood diarrhoea mortality: a country case study

**DOI:** 10.7189/jogh.09.020805

**Published:** 2019-12

**Authors:** Luis Huicho, Mario Tavera, Carlos A Huayanay-Espinoza, Manuel Béjar-Díaz, María Rivera-Ch, Yvonne Tam, Neff Walker, Robert E Black

**Affiliations:** 1 Centro de Investigación en Salud Materna e Infantil, Centro de Investigación para el Desarrollo Integral y Sostenible, Universidad Peruana Cayetano Heredia, Lima, Peru; 2 School of Medicine, Universidad Peruana Cayetano Heredia, Lima, Peru; 3 Departamento de Pediatría, Universidad Nacional Mayor de San Marcos, Lima, Peru; 4 Independent Consultant, Lima, Peru; 5 Johns Hopkins University, Bloomberg School of Public Health, Department of International Health, Institute for International Programs, Baltimore, Maryland, USA

## Abstract

**Background:**

Childhood diarrhoea mortality has declined substantially in Peru in recent decades. We documented trends in childhood diarrhoea mortality from 1980 to 2015, along with trends in coverage of diarrhoea-related interventions and risk factors, to identify the main drivers of mortality reduction.

**Methods:**

We conducted desk reviews on social determinants, policies and programmes, and diarrhoea-related interventions implemented during the study period. We reviewed different datasets on child mortality, and on coverage of diarrhoea-related interventions. We received input from individuals familiar with implementation of diarrhoea-related policies and programmes. We used the Lives Saved Tool (*LiST*) to help explain the reasons for the decline in diarrhoea mortality from 1980 to 2015 and to predict additional reduction with further scale up of diarrhoea-related interventions by 2030.

**Results:**

In Peru under-five diarrhoea mortality declined from 23.3 in 1980 to 0.8 per 1000 livebirths in 2015. The percentage of under-five diarrhoea deaths as related to total under-five deaths was reduced from 17.8% in 1980 to 4.9% in 2015. Gross domestic product increased and poverty declined from 1990 to 2015. Access to improved water increased from 56% in 1986 to 79.3% in 2015. Oral rehydrating salts (ORS) use during an episode of diarrhoea increased from 3.6% in 1986 to 32% in 2015. Vertical programmes focused on diarrhoea management with ORS were implemented successfully in the 1980s and 1990s, and were replaced by integrated crosscutting interventions since the early 2000s. LiST analyses showed that about half (53.9%) of the reduction in diarrhoea mortality could be attributed to improved water, sanitation and hygiene, 25.0% to direct diarrhoea interventions and 21.1% to nutrition. The remaining mortality could be reduced by three-quarters by 2030 with improved diarrhoea treatment and further with enhanced breastfeeding practices and reduction in stunting. *LiST* does not take into account the role of social determinants.

**Conclusions:**

The reduction of diarrhoeal under-five mortality in Peru can be explained by a combination of factors, including improvement of social determinants, child nutrition, diarrhoea treatment with ORS and prevention with rotavirus vaccine and increased access to water and sanitation. The already low rate of diarrhoea mortality could be further reduced by a number of interventions, especially additional use of ORS and zinc for diarrhoea treatment. Peru is a remarkable example of a country that was able to reduce childhood diarrhoea mortality by implementing interventions through vertical programmes initially, and afterwards through implementation of integrated multisectoral packages targeting prevalent illnesses and multi-causal problems like stunting.

Under-five and neonatal mortality rate have decreased remarkably in Peru within the last few decades [1,2], dropping from 125.8 per 1000 live births in 1980 to 16.2 in 2015 [3]. The prevalence of stunted children has also decreased substantially more recently [4]. Previous analyses have explored the possible factors underlying such achievements [1,2].

The proportional contribution of childhood diarrhoea to the total number of under-five deaths has decreased over time at the global level, while the corresponding contribution of neonatal deaths has increased [5]. However, diarrhoea still remains a leading cause of avoidable deaths, particularly in high mortality countries [5]. It was estimated that in 2015 diarrhoea caused about 526 000 under-five deaths [5].

A country level in-depth analysis of factors related to under-five diarrhoea mortality is warranted as an exercise useful to identify reasons for the mortality decline. This can be useful for designing and implementing effective interventions in different regions of the world and within Peru itself, where diarrhoea is still a prevalent condition, particularly in rural areas of the Amazon and the Andes regions.

We aim in this Peru case study to document national trends in diarrhoea mortality from 1980 to 2015, trends in coverage of diarrhoea preventive and curative interventions during the same period, as well as changes in contextual factors and in diarrhoea-related policies and programmes that could have influenced coverage of interventions and diarrhoea mortality. We further model possible changes in interventions and risk factors that could eliminate remaining childhood diarrhoea deaths by 2030.

## METHODS

Our case study relied on a combination of methods that encompassed a desk and literature review, interviews with key informants, evolution of causes of mortality and of interventions coverage over time, and an estimation analysis.

### Desk and literature review

We conducted a desk review of governmental documents and websites, looking for contextual factors and social determinants of health, changes outside the health sector and within the health sector, policies and programmes, and specific diarrhoea-related interventions implemented in Peru over the study period.

To complement the background social, economic and political information, we searched the webpages of diverse organizations involved in studies and technical assessments of contextual aspects of Peru, including the National Institute of Statistics and Computing (INEI), The World Bank, the International Monetary Fund, the Organisation for Economic Co-operation and Development, and the US Congress, by using different combinations of words (“Peru economic history” “Peru economic growth”, “Peru political history”, and “Peru social determinants”).

### Interviews with key informants

We received feedback from individuals familiar with implementation of policies and programmes related to diarrhoeal prevention and control during the study period, to explore possible driving factors that could explain the trend in childhood diarrhoea mortality. We asked them to identify policies and programmes implemented from 1980 to 2015 that may have influenced diarrhoea-related deaths, directly or indirectly, alone or in combination. The participants were identified based on their track record of participation in the design and implementation of the policies, programmes and interventions of interest. They were experts from different sectors (public sector, civil society, academia, multilateral and bilateral organizations and non-governmental organisations). Additional information on diverse implementation aspects of different programmes and interventions was explored through review of technical reports and programmatic documents provided by the interviewees.

The interviews were conducted either in the participants’ workplace or in another agreed ad hoc setting, to ensure their privacy and comfort. The guide topics for the interviews included a) wide crosscutting policies and programmes implemented from 1990 to 2015, b) specific programmes and interventions related to diarrhoea control and management implemented during the study period, and c) main drivers explaining the reduction of childhood diarrhoea mortality. The main assumption of our background theory, which informed the guide topics used, was that the reduction in childhood diarrhoea mortality from 1990 to 2015 in Peru was due to a combination of improvement in contextual factors including social determinants of health, implementation of crosscutting programmes, and implementation of specific diarrhoea-related interventions such as oral rehydrating salts (ORS) utilization during diarrhoeal episodes.

### Time trends of child mortality and coverage of child health interventions

We reviewed information on the evolution of child mortality and its causes from 1980 to 2015 [6], as well as data sets containing information on coverage of out-of-health sector interventions and on specific child health interventions directly or indirectly related to diarrhoea mortality during the same period.

### Estimation of attributed diarrhoea deaths prevented: LiST

The Lives Saved Tool (*LiST*) was used to estimate the possible drivers of the reduction in childhood diarrhoea mortality from 1980 to 2015. *LiST* analysis is based on the estimated efficacy of different interventions in reducing cause-specific mortality or levels of risk factors [7]. Basically, *LiST* models the effects of changes in preventive interventions and in risk factors first, while therapeutic interventions act on the residual diarrheal cases that are not prevented [8].

### Data sources

Diverse data sources were used. Information on economic growth and poverty reduction was obtained from the World Bank [9], from the International Monetary Fund websites ([10]), and from the National Household Surveys (ENAHO) [11]. Data on policies and programmes related to childhood diarrhoea were obtained from the websites of different government and non-government sectors, including those of specific policies and programmes like the conditional cash transfer programme JUNTOS [12], the Comprehensive Health Insurance System [13], the Articulate Nutrition Programme [14], and the Strategic Maternal-Neonatal Programme [15]. Information on the evolution of household coverage of water and sanitation, educational level, and of specific reproductive, maternal, neonatal and child health interventions was obtained from Demographic and Health surveys (DHS) conducted in Peru over the study period [16]. We also looked for unpublished material related to diarrhoea programmes and interventions from the Ministry of Health and UNICEF, particularly for the period of 1980 to 2000, resorting to individuals working currently or having worked in the past in these institutions, as during the mentioned period the probability of finding published policy and programme documents was lower, due to limitations of the country information system for maintaining systematically a complete documentation registry.

We obtained the under-five mortality and the cause-specific deaths from the United Nations Inter-Agency Group for Child Mortality Estimation and the World Health Organization/Maternal and Child Epidemiology Estimation estimates [6].

A list of each variable, the corresponding value and source is provided in Table S1 and Table S2 in **Online Supplementary Document**, with emphasis on input variables for LiST analysis, including coverage targets.

### Data analysis

We constructed time trends for contextual factors, for under-five diarrhoea mortality, and for coverage of diverse interventions related to childhood diarrhoea prevention, control and management. We also constructed a visual illustrating timeline of policies and programmes directly or indirectly related to diarrhoea prevention and control, based on the desk review and on the feedback provided by key informants. We tried to explain the progress of diarrhoea mortality over time within the framework of the changes that occurred in different contextual factors, in policies and programmes, and in the coverage of child health interventions.

We used *LiST* to estimate the attribution of diarrhoea deaths prevented by interventions and changes in nutritional risk factors [8]. The *LiST* modelling approach relies on changes in coverage of interventions of interest over a specified period of time and the efficacy of the intervention to reduce diarrhoea specific under-five mortality. *LiST* also takes into account changes in the prevalence of risk factors such as under-five stunting or wasting and breastfeeding practices, with inclusion of their risk relationship with cause-specific mortality in the model. Additional details on *LiST* as a model for diarrhoea mortality reduction can be found elsewhere [8]. For the *LiST* analysis of the effects of water, sanitation and hygiene (WASH) interventions, the World Health Organization-UNICEF Joint Monitoring Programme was used. This and other sources of data used for the *LiST* analyses can be found in the Table S3 in **Online Supplementary Document**. The time periods of *LiST* analysis of the attribution of diarrhoea mortality reduction to specific interventions and risk factors are 1980-2000 and 2000-2015, as well as the entire 1980-2015. Table S4 in **Online Supplementary Document** lists the set of interventions modelled for these time periods.

For a prospective *LiST* analysis, we modelled universal coverage (90%) of direct and indirect interventions to estimate their potential impact on reducing diarrhoea deaths by 2030, using year 2015 as the baseline. As Peru already has very low rates of wasting and stunting, we have set the targets for these as more ambitious than those of the World Health Assembly. In our analyses we assumed wasting would not be reduced from its 2015 rate of 0.8%. For stunting we are reducing the stunting rate to 8.7% in 2025 which would be a 50% reduction from the stunting rate of 17.3% in 2015. Table S5 in **Online Supplementary Document** lists the set of interventions modelled for this prospective time period. For this prospective *LiST* analysis, we considered three different sets of interventions, which were built as follows. Scenario 1 (direct diarrhoea interventions) is composed by diarrhoea case management through oral rehydration salt solution and zinc, and antibiotic treatment for dysentery. Rotavirus vaccination, a preventive intervention, was also included under scenario 1. Interventions in scenario 2 (nutritional interventions) include improvement in vitamin A coverage, stunting, wasting and breastfeeding, in addition to the interventions in scenario 1. In scenario 3 (WASH interventions), household access to piped water, household access to combined improved drinking and sanitation, and hand-washing with soap were added to the package in scenario 2.

## RESULTS

### Peru political, economic and social background

A democratic government was installed in Peru in 1980, after a period of military dictatorship [17]. From 1980s to 1992, social violence hit Peru, particularly in Andean and Amazon rural areas, and in 2001, after a transient period of political turmoil during the 1990s, democratic stability was regained that has been consolidated over time [17].

As for the evolution of economic aspects, a recent International Monetary Fund report on Peru, distinguishes seven periods from 1976 to 2015, characterized by the oil shock (1976-1984), instability and mismanagement (1985-1990), the great stabilization (1990-1992), sustained recovery (1993-1998), deepening reforms (1999-2007), the global financial crisis (2008-2009), and the post-crisis period (2010 onwards) [10]. In summary, Peru enjoyed a sustained economic growth and stabilisation of the consumer price index since the early 1990s. Real annual growth of average gross domestic product per capita was 0.2% in the period 1961-1990, and reached 3% in the period 1990-2013, being substantially higher relative to the region and the world during the last period [9]. The percentage of the population living in poverty was 46% in the early 80s, 52% in the mid/late 80s, and 58.8% in 1991 (poverty defined as household income that is less than two times the cost of a basic basket of food) [18], illustrating a continued deterioration of the living conditions during this period. According to ENAHO, the proportion of families living with at least one unmet basic need was 53.9% in 1993, 42.1% in 1998, 38.9% in 2001, and this percentage had decreased to 19.7%% in 2014 [19], although there are still substantial segments of the population that remain in poverty, mostly rural areas, with indigenous communities being disproportionately affected, as well as urban pockets of poverty widely scattered across the country [19].

Although the economic and political progress achieved during the last two decades has strengthened the Peruvian social network and the quality of life of citizens, there are still huge challenges to overcome, such as insufficient progress in effective decentralization and low levels of governance and accountability, as crucial bottlenecks in the path to a fully inclusive growth [20-22].

### Time trends of diarrhoea under-five mortality

**Figure 1:**
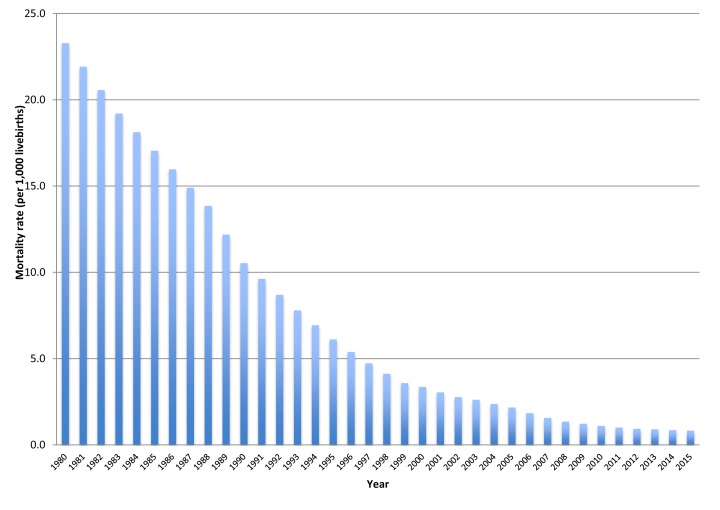
Under-five diarrhoea mortality in Peru, 1980-2015. Source: [6].

The under-five diarrhoea mortality rate in Peru declined dramatically from 23.3 per 1000 livebirths in 1980 (14 551 under five diarrhoea deaths) to 0.8 per 1000 livebirths (3273) in 2015 (**Figure 1**) [6]. The pace of reduction was higher from 23.3 in 1980 to 5.4 per 1000 livebirths 1996, while it declined from 4.7 in 1997 to 0.8 per 1000 livebirths in 2015. Likewise, the percentage of under-five diarrhoea deaths as related to total under-five deaths was reduced from 17.8% in 1980 to 4.9% in 2015 [6].

### Time trend of coverage for diarrhoea-related interventions

The percentage of households with piped water inside the house increased from 56% in 1986 to 79.3% in 2015. The percentage of under-five children who used ORS during an episode of diarrhoea within the 15 days prior to the survey increased from 3.6% in 1986 to 32% in 2015. The household surveys did not collect systematic data on hand washing. Similarly, data were not consistently collected on zinc treatment during diarrhoea episodes. Information about the percentage of children from 6 to 59 months of age who received vitamin A supplementation within the last 6 months was collected only recently. Vitamin A supplementation was incorporated as part of the Growth and Development Monitoring Programme in 2000 [23], while zinc supplementation was introduced officially only in 2017 [24]. This percentage was 7% in 2007 and 6.5% in 2015. Rotavirus vaccination was introduced in Peru in 2007, and national coverage of infants with full course of vaccination progressed from 36.5% in 2009 to 80.9% in 2015. Although the DHS did not provide information about public vs private care-seeking trends, according to programmatic documents from the National Diarrhoea Disease Control Programme, funding sources for this Programme showed a stable trend from the late 1980s to the 1990s. Domestic funding reached US$ 458 000 in 1987 and then 460 000 yearly from 1988 to 19909, while USAID contribution reached US$ 685 000 in 1987 and US$ 700 000 yearly from 1988 to 1990. Meanwhile, UNICEF contribution reached US$ 53 000 in 1987 and US$ 50 000 each year from 1988 to 1990. This illustrates that during the 1980s and 1990s, Peru still relied substantially on external aid to finance implementation of essential public child health programmes. This trend reversed dramatically during the early 2000s, when domestic per capita expenditure on child health increased substantially from US$ 5.6 per child under-five in 2000 to US$ 148.6 in 2012 [25].

### Diarrhoea-related policies and programmes: timeline

**Figure 2:**
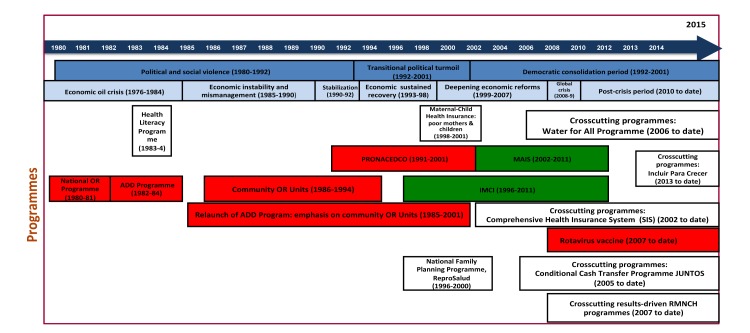
Evolution of diarrhoea-related policies and programmes in Peru. Various sources. OR – oral rehydration, ADD – acute diarrhoeal disease, IMCI – integrated management of childhood illnesses, PRONACEDCO – Prevention and Control of Diarrhoeal Disease Programme, MAIS – Integrated Health Care Model, RMNCH – reproductive, maternal, neonatal and child health. Different colours denote different categories of factors. Social and political context: soft blue. Economic context: light blue. Vertical programmes: red. Integrated programmes: green. Crosscuting programmes: orange-brown.

**Figure 2** summarizes the evolution of diarrhoea-related policies and programmes implemented in Peru from 1980 to 2015 and identified by the interviewees as the most relevant in terms of reduction of diarrhoea-associated mortality.

#### Period 1980-1985: emphasis on case management through oral rehydration

In 1980 The World Health Organization launched at global level the Programme for the Control of Diarrhoeal Diseases, aimed at prioritizing country level implementation, particularly in countries with high child mortality levels caused by diarrhoea, and focused on dehydration management through the use of the ORS at facility and community levels [26]. In a rapid reaction to this global initiative, in Peru the government started in 1980 the implementation of the National Oral Rehydration Programme (*Programa Nacional de Rehidratación Oral*), along with the Breastfeeding Promotion Programme (*Programa de Promocion de Lactancia Materna*) and the Acute Respiratory Infections Control Programme (*Programa de Control de IRA*s).

The top priority intervention of the National Oral Rehydration Programme was the oral rehydration therapy for the management of dehydration cases, aiming at a massive distribution of the oral rehydrating solution sachets (*Bolsita Salvadora)*. Training of health providers was emphasized, along with national production of oral rehydration sachets and then its wide distribution at country level, with support from USAID and UNICEF.

In 1982 the Ministry of Health created the National Diarrhoea Disease Control Programme, which aimed not only to reduce mortality through ORS use, but also to reduce diarrhoea morbidity. Between 1983 and 1984, a Health Literacy Programme was implemented, which promoted actively the use of ORS at community level, through a social marketing strategy that involved local municipalities and private drugstores [27].

Within the global framework of the Child Survival Revolution launched by UNICEF in 1984, which emphasized the use of simple, inexpensive and appropriate technologies to promote the implementation of growth monitoring, oral rehydration, breastfeeding, and immunizations [28], several initiatives to strengthen diarrhoea control efforts were put into place in Peru.

In 1985, a university-based training programme was set up at Universidad Peruana Cayetano Heredia, a leading research university, to train mainly doctors and nurses in the diagnosis and management of common child diseases, including diarrhoea and acute respiratory infections [29]. Shortly afterwards, the first Teaching Oral Rehydrating Unit was set up at Hospital San Bartolomé, a large referral level Ministry of Health hospital in Lima, Peru.

Also in 1985, the national diarrhoeal programme was re-launched with the name of Prevention and Control of Diarrhoeal Disease Programme (*Programa Nacional de Prevención y Control de la Enfermedad Diarreica, PRONACEDCO*) [30], to emphasize that prevention also played an important role in the fight against diarrhoea. Limited progress during the previous 5 years was acknowledged, and specific goals were determined, such as diarrhoeal morbidity and mortality reduction by 20% between 1988 and 1989 through management of 80% of diarrhoeal cases with ORS, expansion of Teaching Oral Rehydration Units in Lima and the rest of the country, improvement of community knowledge and practices related to diarrhoea prevention and management, national production of ORS sachets, and introduction of ORS sachets in private sector drugstores, with active support from USAID.

#### Period 1986-1990

In March 1986, seven children died at the Oral Rehydration Unit from Hospital Cayetano Heredia in Lima, after drinking ORS with excess of potassium, inadvertently prepared. The incident received wide media coverage, and the ORS use declined, albeit temporarily. The same year, the municipality of Lima, in partnership with non-governmental organizations and the Ministry of Health (Control of Diarrhoeal Disease Programme), started the implementation of Community Oral Rehydration Units, within the framework of the Summer Campaign for Diarrhoea Control [31], where key participants were mothers of the Glass of Milk Programme [32].

The role of Community Oral Rehydration Units was strengthened by the Ministry of Health and its partners through various initiatives, although the impact it reached on child mortality has been debated [33]. In 1988, the First Summer Campaign for Diarrhoea Control was launched by the Ministry of Health and UNICEF [34]. This initiative reactivated 1500 Community Oral Rehydration Units, trained 2400 community volunteers, promoted health education through the media, distributed 1 600 000 ORS sachets through Community Oral Rehydration Units and civil society organizations, trained 11 900 additional community volunteers, and implemented 4200 additional Community Oral Rehydration Units across the country. By 1989, there were 2400 active Community Oral Rehydration Units in fourteen departments at national level.

#### 1991-1999 (the cholera epidemic)

The first cholera cases were reported in January 1991 [28]. By September 1991, the number of cases had extended all over the country, with 266 763 probable cases registered.

The case fatality rate during the epidemic was 0.7%, the lowest in Latin America [35].

Although the epidemic reached its peak within the first two years, cholera persisted during the whole decade, prompting the strengthening of diarrhoeal disease-related programmes, strategies and interventions, particularly in the health sector [35]. Prioritized interventions included case management through ORS use, health education campaigns, drinking water chlorination, regulation of food street food vendors, extension of latrines implementation, rehabilitation of school restrooms, and health monitoring of seafood products [34].

In 1994, the Ministry of Health launched the Five-Year Plan for Prevention and Control of Diarrhoeal Diseases (1995-1999). It led to the implementation of 15 000 Community Oral Rehydration Units at national level, to training of 4949 health workers in diarrhoea case management in partnership with the Training of Health Personnel Programme (*Programa de Capacitación en Salud Infantil y Salud de la Mujer*), and to self-sufficiency in the national production of ORS sachets.

In 1999, the Ministry of Health, through *PRONACEDCO*, implemented the last Summer Campaign, within a context of El Niño phenomenon that affected Peru but did not result in a reactivation of the cholera epidemic, although the spring diarrhoeal incidence increased by 55% during El Niño compared with before El Niño [36].

The Integrated Management of Childhood Illness (IMCI) strategy was introduced in Peru in 1996 as an integrated strategy going beyond the previous emphasis on specific child health problems assessed by vertical programmes in a fragmentary way, and it was aimed at further reducing the under-five mortality due to childhood prevalent illnesses such as pneumonia, diarrhoea and malnutrition [37]. It was scaled-up by 1999, reaching virtually all departments of Peru [37]. IMCI was the first national effort to address child health with an integrated approach. Diarrhoea interventions were incorporated as part of IMCI community and facility components. The expansion of IMCI faced several challenges that hampered an adequate and coordinated implementation of its components, reducing therefore its potential for impact on child mortality and nutrition [38].

#### 2000-2015: Integrated approaches and crosscutting programmes

In 2000, vitamin A supplementation for infants younger than 6 months was incorporated as part of the growth monitoring programme [23]. UNICEF donated vitamin A capsules for children under extreme poverty in thirteen priority regions. From 2013 onwards vitamin A capsules were bought by the Ministry of Health directly. There are data showing that under-five vitamin A deficiency in Peru is about 10% [39]. However, it has a low coverage of vitamin A supplementation [39], which is explained, at least partially, by acquisition and supply drawbacks.

In 2002, the Integrated Health Care Model (*Modelo de Atencion Integral de Salud*) strategy was launched (40). Within this context, vertical programmes, including the National Diarrhoea Disease and Cholera Control Programme, ended and some of their activities were integrated as components of childhood interventions,

Building on the previous experience, a Family- and Community-based Integrated Health Care Model (*Modelo de Atención Integral de Salud Basado en Familia y Comunidad)* was implemented since 2011, which consolidated health care as an integrated multisectoral model [40].

The Comprehensive Health Insurance System was implemented in Peru since 2002 and continues to date. It is a subsidized scheme aimed at covering with preventative and curative health services to poor segments of the population, with emphasis on mothers and children under-5, but not limited to them [13]. It coordinates with other initiatives including JUNTOS and other crosscutting programmes [41], providing them with information on the utilization of the available public health services by children and mothers, which. It extended the previous Maternal-Child Health Insurance, which was implemented from 1998 to 2001 and was targeted to poor mothers and children under-5, to receive health services provided by public health facilities. SIS implementation clearly increased maternal and child health services utilization in Peru [42], although an excessive emphasis on curative interventions still needs to be overcome, by introducing adequate incentives to a stronger implementation of promotional and preventative interventions.

The government launched the conditional cash transfer programme JUNTOS in 2005, addressed to the poorest rural families [12]. Under this programme, each eligible family receives PEN 1000 (about US$ 33), conditioned to the use of preventive and curative health services by mothers and children and to school attendance by children of benefited families. Childhood health services include vaccines, micronutrients supplementation, growth monitoring and management of child illnesses, while maternal health services include antenatal care, institutional delivery and postnatal care. The programme is led by the Ministry of Development and Social Inclusion, in coordination with other sectors such as the Ministry of Health and Education [12]. The coverage of rural families with JUNTOS was 1.4% in 2005 and increased quickly until 44.3% in 2013.

The *Water for All Programme* has been implemented by the Peruvian government since 2006 [43]. It was led by the Ministry of Housing, Construction and Sanitation, along with the Ministry of Health that was in charge of hygiene promotion initiatives, the National Superintendence of Sanitation Services as the supervisory body, and local governments that are often developers and managers [43]. *Water for All* attempted to increase access to safe water and sanitation for the poorest segments of the population. An independent evaluation conducted in 2009 concluded that implementation of *Water for All* had various programmatic flaws that hampered its impact, including discordance between its strategic objective and the effective targeting of the poorest, an excessive focus on infrastructure, neglect of social management to guarantee sustainable utilization of water and sanitation facilities built, and lack of specific efficacy indicators [44]. In 2016 the Project *Agua Más* was introduced, which is an intervention led by the Ministry of Inclusion and Social Development, aimed at rehabilitating, replacing and ensuring the functioning and maintenance of water and sanitation systems in the rural areas of Peru, to reduce in this way the infrastructure quality gap and to ensure access to safe water and sanitation to the poorest segments of the population [45].

Since 2007, crosscutting programmes aimed at reducing child stunting and maternal and neonatal mortality through multisectoral interventions were launched and scaled up by the government, with the leadership of the Ministry of Economy and Finances and the active participation of the Ministry of Health and other involved sectors [14,15]. One of these initiatives, the Articulate Nutrition Programme, aimed at reducing infant and child stunting and anaemia, involves the implementation of crosscutting interventions ranging from social determinants of health to out-of-health-sector changes to health sector changes to proximal factors namely specific reproductive, maternal, neonatal and child health interventions health interventions known to reduce malnutrition [14], including prevention and management of diarrhoeal diseases. The other one, the Strategic Maternal-Neonatal Programme, aimed at reducing maternal and neonatal mortality, is focused on increasing coverage of interventions such as family planning and modern contraception, appropriate antenatal care, institutional delivery, skilled birth attendant, and postnatal care of mothers and newborns [15].

It is within this new wide multisectoral approach context that new specific child health interventions were implemented, among them the rotavirus vaccine, introduced in Peru in 2007 [46].

#### *LiST* analyses

The under-five diarrhoeal-specific mortality rate (DSMR) in Peru, expressed in percentage of overall under-five mortality, was 17.8 in 1980, 8.6 in 2000, and 4.9 in 2015, with a per cent reduction of 72.4% [6]. The *LiST* analysis showed changes in stunting and wasting and coverage accounted for 76% of the estimated overall reduction in diarrhoea mortality from the global analyses reported in Liu et al. [5].

The percentage of diarrhoea mortality reduction attributable to different factors by time period is shown in Table 1. Comparing 1980 to 2015, the percentages of DSMR reduction attributable to changes in direct diarrhoea interventions, nutrition, and WASH interventions were 25.0%, 21.1% and 53.9%, respectively. Corresponding figures comparing 1980 to 2000 were 15.1%, 23.6% and 61.3%, respectively, and for the comparison of 2000 to2015 they were 45.9%, 13.7% and 40.4%, respectively. The single intervention with the greatest impact attribution in the overall period and in each sub-period was household access to piped water ((30.4% in 1980-2015, 34.7% in 1980-2000 and 26.2% in 2000-2015). Likewise, a substantial proportion of the reduction could be attributed to ORS especially in the first and second periods (14.2% and 14.5%, respectively), and to rotavirus vaccination in the second period (25.6% of the reduction). The contribution of stunting reduction was also important, particularly in the first period (16.2% of the overall reduction). The number of diarrhoea deaths averted is shown in Table S6 in the **Online Supplementary Document**. Note these values are not cumulative but rather the number of deaths averted in a single year.

**Table 1:** Percent of under-five diarrhea mortality reduction attributable to different interventions in Peru, first to last year in a time period

Interventions	1980-2000 Attribution	2000-2015 Attribution	1980-2015 Attribution
Zinc for treatment of diarrhea*	0.0%	0.0%	0.0%
Rotavirus vaccine*	0.0%	25.6%	12.8%
ORS*	14.2%	14.5%	9.8%
Antibiotics for dysentery*	0.8%	0.0%	0.3%
Persistent diarrhea treatment*	0.0%	5.9%	2.1%
Changes in age-appropriate breastfeeding practices†	4.9%	0.0%	2.3%
Early initiation of breastfeeding†	0.2%	0.0%	<0.1%
Vitamin A supplementation†	0.3%	0.1%	0.2%
Changes in stunting prevalence†	16.2%	11.6%	16.8%
Changes in wasting prevalence†	2.1%	2.0%	1.7%
Combination of improved water source and improved sanitation‡	13.7%	7.3%	12.0%
Water connection in the home‡	34.7%	26.2%	30.4%
Hand washing with soap‡	13.0%	6.9%	11.4%
Total Scenario 1 (Direct*)	15.1%	45.9%	25.0%
Total Scenario 2 (Direct* + Nutrition†**)**	38.7%	59.6%	46.1%
Total Scenario 3 (Direct* + Nutrition† **+ WASH**‡**)**	100.0%	100.0%	100.0%

ORS – oral rehydration solution, WASH – water, sanitation and hygiene

*Direct diarrhoea interventions.

†Nutritional interventions.

‡WASH interventions.

The estimations of under-five diarrhoea mortality reduction percentage attributable to different interventions by scenario for the period 2015-2030 are shown in **Table 2**. Assuming universal coverage (90%) for the different interventions including exclusive breastfeeding and 50% reduction in under-five stunting by year 2025, the DSMR in Peru would drop to 0.21 under scenario 1 (direct diarrhoea interventions only), to 0.13 under scenario 2 (direct diarrhoea plus nutritional interventions) and to 0.12 under scenario 3 (direct diarrhoea, nutritional interventions, hand washing and improved sanitation/water interventions). ORS would be the major contributor under all scenarios (58% under scenario 1, 34% under scenario 2 and 30.4% under scenario 3). The percentages of diarrhoea deaths averted are available in Table S7 in the **Online Supplementary Document**.

**Table 2:** Percent of under-five DSMR reduction attributable to different interventions (%), Peru, 2015-2030, by scenario

Interventions	Scenario 1 (Direct*)	Scenario 2 (Direct* + nutrition†)	Scenario 3 (Direct* + nutrition† + WASH‡)
Zinc for treatment of diarrhea*	19.9%	11.4%	10.3%
Rotavirus vaccine*	0.9%	0.7%	0.7%
ORS*	58.0%	34.0%	30.4%
Antibiotics for dysentery*	8.2%	4.9%	4.3%
Persistent diarrhoea treatment*	13.1%	7.5%	6.7%
Changes in age-appropriate breastfeeding practices†		18.7%	17.2%
Vitamin A supplementation†		5.3%	5.0%
Changes in stunting prevalence†		17.5%	16.3%
Combination of improved water source and improved sanitation‡			5.0%
Hand washing with soap‡			4.1%
**Total**	100.0%	100.0%	100.0%

DSMR – diarrhoeal specific mortality rate, ORS – oral rehydration salts

*Direct diarrhoea interventions.

†Nutritional interventions.

‡WASH interventions.

## DISCUSSION

The progress achieved by Peru in the reduction of under-five mortality and in the reduction of under-five stunting can be explained as the result of a combination of factors, namely improvement in social determinants of health, significant out-of-health and within health sector changes, political leadership, strong civil societal advocacy, and equitable implementation of child interventions [1,2,4]. The greatest proportion of such reduction is accounted by reduction in pneumonia, diarrhoea and neonatal deaths [1,2].

Of note, the greatest reduction of under-five diarrhoea mortality rate in Peru, from 23.3 per 1000 livebirths in 1980 to 5.4 in 1996, coincided with the implementation of vertical childhood programmes, which is a testimony of the effectiveness of specific interventions like the use of ORS in the diarrhoea case management.

Considering the evolution of diarrhoea-related policies and programmes since 1980, a focus on implementation of diarrhoea-specific interventions in the 1980s and 1990s is clear. Such interventions were initially directed to the management of dehydration through the promotion of ORS, followed shortly by interventions addressed at both prevention and case management. These were implemented as part of vertical programmes with clear coverage, budget line, training and supervision targets, which resulted in increases in ORS use at facility and community levels [47]. These implementation strengths can explain at least in part the reduction of diarrhoea mortality and the low case fatality rate even in a very large cholera epidemic in Peru [35].

Then the country turned its attention to integrated approaches, which allowed a holistic assessment of children going beyond the isolated approach to specific childhood illnesses. IMCI was introduced in 1996, with efforts to scale up that were intensified during the 2000s [37]. Although IMCI was adopted quickly as a novel integrated strategy, it could not be scaled up successfully due to weak political leadership and commitment, lack of a separate budget line, uncoordinated geographical implementation of its clinical and community components, lack of effective health system strengthening activities, overlapping and competition with vertical programmes, and scattered implementation that was not clearly focused on the poorest regions of the country [37,38].

Of note, a few interventions shown to be effective to reduce childhood diarrhoea mortality have not been implemented in Peru or have been implemented only at a minimal scale. For vitamin A and zinc, their almost zero coverage can be due, at least partially, to the shift from vertical to integrated approaches to child illness that seems to have reduced the emphasis on ORS use at facility and community level [38]. Likewise, neither vitamin A nor zinc implementation were prioritized under these new approaches. Efficient purchase and distribution mechanisms were also neglected. Zinc has been included only in 2017 as part of the national paediatric diarrhoea treatment guidelines [24]. Interestingly, the Peruvian DHS data show consistently over time that a high proportion of children consume vitamin A-rich food [47], which could suggest that the additional benefit of vitamin A in such a context would be limited.

Since the early 2000s, a multisectoral approach to tackle maternal and child health and nutrition problems was introduced with renewed impetus in Peru [48]. It was aimed at poverty reduction and improvement of other social determinants of health [12], along with out-of-health and within-health sector changes, as well as implementation of specific maternal and child health interventions including those aimed at prevention and management of diarrhoea and under-nutrition reduction within this broad crosscutting perspective [14,15,49]. One prominent example includes the conditional cash transfer JUNTOS that was introduced in 2005 and was scaled-up quickly afterwards [12], to reduce the poverty rate in rural areas of the Amazon and the Andes. It increased household consumption and income, increased the utilization of curative and preventive health services, and it is likely that played a role in the continued decrease of DSMR in Peru, probably through the reduction of childhood malnutrition, as there is evidence of its positive effect on longitudinal child growth [50]. The implementation of the Comprehensive Health Insurance System since 2002, which was associated with a substantial increase in health services utilization by mothers and children [51], may have also contributed to the reduction of maternal and child mortality, although there are still challenges for overcoming health system limitations such as remaining inequities [42]. We must also highlight the implementation of crosscutting results-based programmes, like the Articulate Nutrition Programme and the Strategic Maternal-Neonatal Programme, the first aimed at further reducing under-5 stunting prevalence, diarrhoea, pneumonia and other prevalent childhood diseases, and the second aimed at further reducing maternal and neonatal mortality. Under these programmes, the Ministry of Economy and Finances allocated the funds to regional and local governments, based on specific results showed in terms of progress reached in the coverage of the included interventions and in the reduction of targeted outcomes, namely under-5 and under-3 stunting prevalence, anaemia prevalence, and maternal and neonatal mortality [52,53].

Clearly, the deactivation of vertical programmes and the increasing adoption of multisectoral integrated programmes in Peru were associated with positive and negative aspects. While the initial emphasis on vertical programmes allowed their sustained implementation with clear coverage goals, specific budget lines and dedicated human resources targeted to specific problems like acute diarrhoeal and respiratory diseases, they showed limitations for tackling social determinants of health and other contextual problems intimately related to complex problems like child stunting, and were not necessarily appropriate for facilitating multisectoral collaboration. Conversely, while the integrated multisectoral programmes widely adopted in more recent times proved to be part of a powerful approach to tackle multi-causal problems like child malnutrition through collaboration with multiple sectors [1,4], their implementation run in parallel with that of vertical programmes, at least initially, and they competed frequently for limited financial resources [38], with the consequent decreasing priority and even the abandonment of vertical programmes.

Under all scenarios of universal implementation of different interventions, by 2030 the DSMR would drop in Peru even further below 1 per 1000 live births. Even if Peru has already achieved substantial improvement in coverage of most interventions, further increase in ORS and zinc treatment, antibiotics for dysentery and treatment for persistent diarrhoea would still result in substantial reduction of all under-five diarrhoeal deaths in the future. Rotavirus vaccine would have a rather limited additional impact on DSMR, because coverage is already high. Nutritional interventions added to direct diarrhoea interventions that would further decrease DSMR by 2030 including changes in age-appropriate breastfeeding practices, changing in stunting prevalence and vitamin A supplementation. Conversely, increase in early initiation of breastfeeding would have little effect because diarrhoea is a very infrequent cause of neonatal deaths. Changes in wasting prevalence would not have an additional impact because wasting prevalence is already below 1% in Peru. It is important to remark that our *LiST* analyses do not capture the role of social determinants of health, and therefore alternative approaches may be needed to address the influence of such aspects on DSMR.

These analyses may be a useful guide to design and implement future interventions at national level. They need to be taken into account within the wider Peruvian context, where under-five diarrhoea mortality is already low and stunting has also declined rapidly to 14.4% in 2015, although there are still substantial gaps between urban and rural areas and between rich and poor segments of the population, both for interventions coverage and for impact indicators. Stunting reduction, an important driver of further DSMR, has been achieved in Peru thanks to the sustained implementation of crosscutting, pro-poor, evidence-based interventions enabled by strong political leadership and civil society advocacy [4]. At the same time, further substantial diarrhoea mortality gains can also occur with universal implementation of direct diarrhoea interventions like zinc treatment, ORS, antibiotics for dysentery and persistent diarrhoea treatment, dependent on a functional health system able to provide quality health services. It is likely that design and implementation of different packages of interventions will be needed, which should be customized to specific geographic, economic and cultural characteristics of different regions of the country, particularly of the rural Amazon and Andean regions, where diarrhoea is still a leading cause of under-five mortality.

In conclusion, the reduction of diarrhoeal under-five mortality in Peru can be explained by a combination of factors, including improvement of social determinants, child nutrition, and increased access to public health services such as water and sanitation, along with an increase in the coverage of interventions delivered by the health system such as ORS for dehydration, antibiotics for dysentery, and rotavirus vaccination. Vertical diarrhoea control programmes in the 1980s and 1990s focused on dehydration management with ORS were successfully implemented at facility and community levels, and prepared the Peruvian health system to face effectively the cholera epidemic that hit Peru in the 1990s [35]. They were later replaced by integrated crosscutting multisectoral interventions, which were effective in further improving child survival and nutrition [2,4]. Peru is a remarkable example of a country that was able to reduce childhood diarrhoea mortality by implementing effective interventions through vertical programmes initially, and afterwards through the implementation of integrated multisectoral packages of interventions addressed both at reducing mortality due to prevalent illnesses and at reducing complex multi-causal problems like stunting.

## Additional material

Online Supplementary Document
