# Supplementary material for: Drivers of the progress achieved by Peru in reducing childhood diarrhoea mortality: a country case study
Source: J Glob Health. 2019 Sep 22;9(2):020805. doi: 10.7189/jogh.09.020805 (PMC6816317; doi:10.7189/jogh.09.020805)
Supplement: Online Supplementary Document [file jogh-09-020805-s001.pdf]

Huicho et al. Drivers of the progress achieved by Peru in reducing childhood diarrhoea mortality: A country case study

Online Supplementary Material

Table S1

Coverage and data sources of interventions used in LiST analyses (1980-2015).

Intervention	Age Range	1980 Coverage/ Prevalence (%)	Earliest Measured Data (%)	2000 Coverage/ Prevalence (%)	2015 Coverage/ Prevalence (%)	2030 Target Coverage/ Prevalence (%)	Data Source Notes
Zinc for treatment of diarrhea		0.0	0.0	0.0	0.0	90.0	No data on zinc for treatment of diarrhea in Peru.
Rotavirus vaccine		0.0	3.0 (WHO/UNICEF 2008)	0.0	87.0 (WHO/UNICEF 2015)	90.0	
ORS		0.0	20.7 (DHS 1991)	21.9 (Interpolated)	28.2 (DHS 2014)	90.0	Assumed coverage was 0 in 1980 and used linear interpolation between 1980 and earliest measured data.
Antibiotics for dysentery		2.7 (Interpolated)	20.5 (DHS 1991)	17.3 (DHS 2000)	16.0 (DHS 2014)	90.0	Assumed coverage was 0 in 1960 and used linear interpolation between 1960 and earliest measured data.
Persistent diarrhea treatment		0.0	0.0	0.0	33.0	90.0	No data on persistent diarrhea treatment in Peru. Coverage in 2015 based on expert opinion.
Changes in age-appropriate breastfeeding	0-1 months (exclusive breastfeeding)		77.8 (DHS 1996)	85.6 (DHS 2000)	68.9 (DHS 2012)	90.0	
	1-5 months (exclusive breastfeeding)		50.6 (DHS 1996)	65.2 (DHS 2000)	67.5 (DHS 2012)	90.0	
	6-11 months (any breastfeeding)		90.3 (DHS 1996)	94.7 (DHS 2000)	92.0 (DHS 2012)	92.0	
	12-24 months (any breastfeeding)		65.0 (DHS 1996)	68.0 (DHS 2000)	65.8 (DHS 2012)	90.0	
Early initiation of breastfeeding		40.2 (Assumed equal to earliest measured data)	40.2 (DHS 1996)	44.9 (DHS 2000)	53.7 (DHS 2012)	90.0	Assumed 1980 coverage equal to earliest measured data. Intervention not scaled
Vitamin A supplementation		0.0	3.78 (DHS 2000)	3.78 (DHS 2000)	4.5 (DHS 2012)	90.0	Assumed 1990 year of introduction. Used linear interpolation between 1990 and earliest measured data
Changes in stunting prevalence		38.2 (Majid et al)	31.1 (DHS 1996)	30.5 (Interpolated)	17.3 (DHS 2013)	8.7	Overall stunting rates obtained from Majid *et al.* Age structure from earliest measured data and modeled overall stunting rate used to

							calculate 1980 stunting distributions, segregated by age.
Changes in wasting prevalence		1.8 (1991 DHS)	1.4 (DHS 1996)	1.1 (Interpolated)	0.5 (DHS 2013)	-	
Improved water source		71.7 (Interpolated)	77.7 (JMP 1996)	79.8 (JMP 2000)	86.7 (JMP 2015)	90.0	Assumed coverage was 0 in 1960 and used linear interpolation between 1960 and earliest measured data.
Improved sanitation		48.6 (Interpolated)	59.0 (JMP 1996)	62.9 (JMP 2000)	76.2 (JMP 2015)	90.0	Assumed coverage was 0 in 1960 and used linear interpolation between 1960 and earliest measured data.
Water connection in the home		33.0	60.8 (JMP 1996)	64.7 (JMP 2000)	78.2 (JMP 2015)	-	
Hand washing with soap		33.0	60.8 (JMP 1996)	64.7 (JMP 2000)	78.2 (JMP 2015)	90.0	Assumed coverage equal to coverage of water connection in the home in 1980 and linear scale up to earliest measured data from JMP

Table S2
Interventions' coverage (%) between years 2015 to 2030.

Interventions	Data source for 2015 coverage	Coverage (%) 2015	2016	2017	2018	2019	2020	2021	2022	2023	2024	2025	2026	2027	2028	2029	2030
Zinc for treatment of diarrhea[1]	No data for this intervention in Peru	0.0	6.0	12.0	18.0	24.0	30.0	36.0	42.0	48.0	54.0	60.0	66.0	72.0	78.0	84.0	90.0
Rotavirus vaccine[1]	WHO/UNICEF	87.0	87.2	87.4	87.6	87.8	88.0	88.2	88.4	88.6	88.8	89.0	89.2	89.4	89.6	89.8	90.0
ORS[1]	DHS	37.4	40.9	44.4	47.9	51.4	54.9	58.4	61.9	65.5	69.0	72.5	76.0	79.5	83.0	86.5	90.0
Antibiotics for dysentery[1]	DHS	16.0	20.9	25.9	30.8	35.7	40.7	45.6	50.5	55.5	60.4	65.3	70.3	75.2	80.1	85.1	90.0
Persistent diarrhoea treatment[1]	Based on expert opinion	33.0	36.8	40.6	44.4	48.2	52.0	55.8	59.6	63.4	67.2	71.0	74.8	78.6	82.4	86.2	90.0
Changes in age-appropriate breastfeeding practices[2]																	
Exclusive breastfeeding; <1 month	DHS	68.9	70.3	71.7	73.1	74.5	75.9	77.4	78.8	80.2	81.6	83.0	84.4	85.8	87.2	88.6	90.0
Predominant breastfeeding; <1 month		4.8	4.8	4.8	4.8	4.8	4.8	4.8	4.8	4.8	4.8	4.8	4.8	4.8	4.8	4.8	4.8
Partial breastfeeding; <1 month		22.5	21.0	19.6	18.2	16.8	15.4	14.0	12.6	11.2	9.8	8.4	7.0	5.6	4.2	2.8	1.4
Not breastfeeding; <1 month		3.8	3.8	3.8	3.8	3.8	3.8	3.8	3.8	3.8	3.8	3.8	3.8	3.8	3.8	3.8	3.8

Exclusive breastfeeding; 1-5 months		67.5	69.0	70.5	72.0	73.5	75.0	76.5	78.0	79.5	81.0	82.5	84.0	85.5	87.0	88.5	90.0
Predominant breastfeeding; 1-5 months		4.8	4.8	4.8	4.8	4.8	4.8	4.8	4.8	4.8	4.8	4.8	4.8	4.8	4.8	4.8	4.8
Partial breastfeeding; 1-5 months		25.2	23.7	22.2	20.7	19.2	17.7	16.2	14.7	13.2	11.7	10.2	8.7	7.2	5.7	4.2	2.7
Not breastfeeding; 1-5 months		2.5	2.5	2.5	2.5	2.5	2.5	2.5	2.5	2.5	2.5	2.5	2.5	2.5	2.5	2.5	2.5
Any breastfeeding; 6-11 months		92.0	92.0	92.0	92.0	92.0	92.0	92.0	92.0	92.0	92.0	92.0	92.0	92.0	92.0	92.0	92.0
Not breastfeeding; 6-11 months		8.0	8.0	8.0	8.0	8.0	8.0	8.0	8.0	8.0	8.0	8.0	8.0	8.0	8.0	8.0	8.0
Any breastfeeding; 12-23 months		65.8	67.4	69.0	70.6	72.3	73.9	75.5	77.1	78.7	80.3	81.9	83.5	85.2	86.8	88.4	90.0
Not breastfeeding; 12-23 months		34.2	32.6	31.0	29.4	27.7	26.1	24.5	22.9	21.3	19.7	18.1	16.5	14.8	13.2	11.6	10.0
Vitamin A supplementation[2]	UNICEF	4.5	10.2	15.9	21.6	27.3	33.0	38.7	44.4	50.1	55.8	61.5	67.2	72.9	78.6	84.3	90.0
Changes in stunting prevalence[2]	DHS	17.3	17.3	17.3	16.2	15.2	14.1	13.0	11.9	10.8	9.7	8.7	8.7	8.7	8.7	8.7	8.7
Combination of Improved Water Source and Improved Sanitation[3]	JMP	76.2	77.1	78.0	79.0	79.9	80.8	81.7	82.6	83.6	84.5	85.4	86.3	87.2	88.2	89.1	90.0

Hand washing with soap[3]	DHS - used coverage of water connection in the home as a proxy	78.2	79.0	79.8	80.6	81.4	82.2	82.9	83.7	84.5	85.3	86.1	86.9	87.6	88.4	89.2	90.0

Table S3
Data source documentation.

Indicator	Source(s)	Assumptions
Neonatal, infant and under-five mortality rates	United Nations Population Division	None
Height-for-age Z score (HAZ) distribution data, segregated by age group	Overall stunting rates (% of children under five greater than -2 SD below reference population mean and % of children under five greater than -3SD below reference population mean) for year 1985[58]<br><br>When available, data from nationally representative surveys were used*	Modeling in LiST required Stunting distribution pattern by age. Matched stunting distribution by age from earliest DHS, or similar survey. Assumed 1980 stunting distribution equal to 1985 stunting distribution[58]
Weight-for-age Z score (WHZ) distribution data, segregated by age group	Nationally representative surveys*	Assumed 1980 coverage equal to earliest measured from nationally representative surveys. For subsequent years, linear changes were assumed between measured values from nationally representative surveys
Cause of death structure (i.e. percentage of deaths due to each major cause of death)	Maternal Child Epidemiology Estimation	None
Antibiotics for dysentery	Nationally representative surveys*	Coverage equal to 0% in year 1960and linear scale up until earliest measured data
Early Initiation of breastfeeding	Demographic and Health Surveys, Multiple Indicator Cluster Surveys, other national surveys	Assumed 1980 coverage equal to earliest measured
Hand washing with soap	Joint Monitoring Programme	Assumed coverage equal to coverage of water connection in the home in 1980 and linear scale up to earliest measured data from JMP or 2009 global estimate
Improved sanitation - Utilization of latrines or toilets + improved water source	Joint Monitoring Programme	Assumed coverage equal to 0% in year 1960 and linear scale up until earliest measured data. Effectiveness applies only when improved sanitation and improved water are both present.
Rotavirus vaccine (two doses)	UNICEF vaccine coverage data	Assumed 0% unless measured data available
Oral rehydration solution	Nationally representative surveys*	Assumed coverage equal to 0% in year 1980 and linear scale up until earliest measured data
Vitamin A supplementation	UNICEF Vitamin A supplementation data	Assumed overage equal to 0% in 1980. Assumed coverage equal to 0% in year 1990 and linear scale up until earliest measured data
Persistent diarrhea	None	Assumed coverage equal to 0% in 2000 and 33% in all countries

*DHS, MICS surveys and other similar surveys. Please note that in Peru there were 10 DGS surveys between 1986 and 2012. Since that time Peru has been running continuous surveys and therefore has yearly estimates of stunting, wasting and coverage of interventions.

Table S4

Description of the interventions and risk factors included in each universal coverage scale-up scenario for years 1980 to 2015.

	Scenario 1<br>Direct Diarrhea Interventions	Scenario 2<br>Direct Diarrhea Interventions and Nutrition Interventions	Scenario 3<br>All interventions that affect diarrhea mortality
Interventions scaled up to universal coverage (90%)<br><br>(For stunting, target = reduction by 50%, for wasting target= <5%)	• Antibiotics for dysentery<br>• Oral rehydration solution<br>• Persistent diarrhea treatment<br>• Rotavirus vaccine<br>• Zinc	• All direct diarrhea interventions<br>• Vitamin A<br>• Stunting prevalence<br>• Wasting prevalence<br>• Breastfeeding practices<br>• Early initiation of breastfeeding	• All direct diarrhea interventions<br>• All nutrition interventions<br>• Improved sanitation + improved water source<br>• Water connection in the home<br>• Handwashing

Table S5

Description of the interventions and risk factors included in each universal coverage scale-up scenario for years 2015 to 2030.

	Scenario 1<br>Direct Diarrhea Interventions	Scenario 2<br>Direct Diarrhea Interventions and Nutrition Interventions	Scenario 3<br>All interventions that affect diarrhea mortality
Interventions scaled up to universal coverage (90%)<br><br>(For stunting, target = reduction to 8.7% in year 2025)	• Antibiotics for dysentery<br>• Oral rehydration solution<br>• Persistent diarrhea treatment<br>• Rotavirus vaccine<br>• Zinc	• All direct diarrhea interventions<br>• Vitamin A<br>• Stunting prevalence<br>• Breastfeeding practices	• All direct diarrhea interventions<br>• All nutrition interventions<br>• Improved sanitation + improved water source<br>• Handwashing

Table S6
Number of diarrhea deaths reduction attributable to different interventions in Peru comparing first to last year in a time period.

Interventions	1980-2000 Attribution	2000-2015 Attribution	1980-2015 Attribution
Zinc for treatment of diarrhea[1]	0	0	0
Rotavirus vaccine[1]	0	292	1,480
ORS[1]	1,225	165	1,135
Antibiotics for dysentery[1]	72	0	34
Persistent Diarrhoea Treatment[1]	0	67	239
Changes in age-appropriate breastfeeding practices[2]	418	0	269
Early initiation of breastfeeding[2]	16	0	0
Vitamin A supplementation[2]	25	1	24
Changes in stunting prevalence[2]	1,395	132	1,935
Changes in wasting prevalence[2]	179	23	201
Combination of Improved Water Source and Improved Sanitation[3]	1,176	83	1,385
Water connection in the home[3]	2,983	299	3,510
Hand washing with soap[3]	1,119	79	1,317
**Total Scenario 1 (Direct[1])**	1,297	524	2,888
**Total Scenario 2 (Direct[1] + Nutrition[2])**	3,330	680	5,317
**Total Scenario 3 (Direct[1] + Nutrition[2] + WASH[3])**	8,608	1,141	11,529

Table S7
Percent of DSMR reduction attributable to different interventions (%), Peru, 2015-2030, by scenario.

Interventions	Scenario 1 (Direct[1])	Scenario 2 (Direct[1] + Nutrition[2])	Scenario 3 (Direct[1] + Nutrition[2] + WASH[3])
Zinc for treatment of diarrhea[1]	70	47	43
Rotavirus vaccine[1]	3	3	3
ORS[1]	204	140	127
Antibiotics for dysentery[1]	29	20	18
Persistent Diarrhoea Treatment[1]	46	31	28
Changes in age-appropriate breastfeeding practices[2]		77	72
Vitamin A supplementation[2]		22	21
Changes in stunting prevalence[2]		72	68
Combination of Improved Water Source and Improved Sanitation[3]			21
Hand washing with soap[3]			17
**Total**	352	412	418
